# Enhancement of Farnesoid X Receptor Inhibits Migration, Adhesion and Angiogenesis through Proteasome Degradation and VEGF Reduction in Bladder Cancers

**DOI:** 10.3390/ijms23095259

**Published:** 2022-05-09

**Authors:** Chien-Rui Lai, Hisao-Hsien Wang, Hsin-Han Chang, Yu-Ling Tsai, Wen-Chiuan Tsai, Chen-Ray Lee, Chih-Ying Changchien, Yu-Chen Cheng, Sheng-Tang Wu, Ying Chen

**Affiliations:** 1 Department of Biology and Anatomy, National Defense Medical Center, Taipei 11490, Taiwan; ray42904917@gmail.com (C.-R.L.); albertchang1008@gmail.com (H.-H.C.); koala8072@yahoo.com.tw (C.-Y.C.); plokmijzz@gmail.com (Y.-C.C.); 2 Department of Urology, Cheng Hsin General Hospital, Taipei 11490, Taiwan; hsiao386@ms13.hinet.net; 3 Department of Pathology, Tri-Service General Hospital, National Defense Medical Center, Taipei 11490, Taiwan; c909228@gmail.com (Y.-L.T.); ab95057@hotmail.com (W.-C.T.); 4 Department of Medicine, National Defense Medical Center, Taipei 11490, Taiwan; edwin890523@gmail.com; 5 Department of Internal Medicine, Tri-Service General Hospital, National Defense Medical Center, Taipei 11490, Taiwan; 6 Division of Urology, Department of Surgery, Tri-Service General Hospital, National Defense Medical Center, Taipei 11490, Taiwan

**Keywords:** FXR, bladder cancer, migration, invasion, angiogenesis, VEGF, proteasomal degradation, protein half-life

## Abstract

(1) Background: Bladder cancer is a malignant tumor mainly caused by exposure to environmental chemicals, with a high recurrence rate. NR1H4, also known as Farnesoid X Receptor (FXR), acts as a nuclear receptor that can be activated by binding with bile acids, and FXR is highly correlated with the progression of cancers. The aim of this study was to verify the role of FXR in bladder cancer cells. (2) Methods: A FXR overexpressed system was established to investigate the effect of cell viability, migration, adhesion, and angiogenesis in low-grade TSGH8301 and high-grade T24 cells. (3) Results: After FXR overexpression, the ability of migration, adhesion, invasion and angiogenesis of bladder cancer cells declined significantly. Focal adhesive complex, MMP2, MMP9, and angiogenic-related proteins were decreased, while FXR was overexpressed in bladder cancer cells. Moreover, FXR overexpression reduced vascular endothelial growth factor mRNA and protein expression and secretion in bladder cancer cells. After treatment with the proteosome inhibitor MG132, the migration, adhesion and angiogenesis caused by FXR overexpression were all reversed in bladder cancer cells. (4) Conclusions: These results may provide evidence on the role of FXR in bladder cancer, and thus may improve the therapeutic efficacy of urothelial carcinoma in the future.

## 1. Introduction

Bladder cancer is the tenth most common cancer worldwide, and it ranks as the sixth most common cancer and the ninth leading cause of cancer death among men. In 2020, nearly 575,000 new cases were diagnosed [1]. In Taiwan, bladder cancer is the ninth most common cancer among men (Centers for Disease Control, Ministry of Health and Welfare, Taiwan, 2020). Approximately, 75% of patients showed a non-muscle-invasive type, while others showed a muscle-invasive type, frequently with metastasis [2]. The mortality rates have been declining, mainly due to the improvements in treatment (e.g., endoscopic resection, adjuvant instillation of chemotherapy, and intravesical immunotherapy) [3]. Previous studies have shown that the existence of some compounds that could have a protective role against bladder cancer, including kaempferol, fisetin, and myricetin [4]. In addition, the detection of clinical biomarkers NMP22, MDX, and uCAPP in urine are also new therapeutic strategies explored in recent studies [5]. However, the recurrence rate in bladder cancer patients is still an serious issue. According to the statistics, nearly 75% of patients diagnosed with bladder cancer will recur or progress within ten years [6]. Above all, increasing efforts should be put towards offering bladder cancer patients some new therapeutic strategies, which is a critical issue in bladder cancer research.

The farnesoid X receptor (FXR, encoded by the NR1H4 gene) functions as a bile acid nuclear receptor [7], and is expressed mainly in the liver, intestine, kidney, and adrenal glands [8]. After being activated by ligands, FXR interacts with its heterodimer partner retinoid X receptor (RXR) and binds to FXR response element (FXRE). Next, FXR modulates the expression of the downstream target genes, including bile acid homeostasis [9], fatty acid metabolism [10], and glucose metabolism [11]. Moreover, FXR induces gene expression of small heterodimer partner (SHP), which suppresses the expression of CYP7A1 and reduces hepatic bile acid synthesis via negative feedback in cholesterol metabolism [12].

Recently, the expression and role of FXR in cancers have been investigated. In breast cancer, high expression of FXR was reported and shown to induce bone metastasis by activating the expression of runt-related transcription factor (RUNX2) and mimicking the bone microenvironment [13]. In addition, Caco2 and HT29 colon cancer cells show a high expression of FXR, which contributes to cell differentiation and proliferation [14]. However, FXR has also shown an opposite role in cancers. Lower FXR expression is associated with higher tumor grade in colon cancer [14]. Moreover, patients have shown decreased expression of FXR in tumors compared to the normal colon tissues. In colorectal cancer, overexpressing FXR decreases the proliferation of cancer stem cells, thereby inhibiting cancer carcinogenesis [15]. In cervical cancer, FXR overexpression inhibits cervical squamous carcinoma cell proliferation via the upregulation of SHP, MDM2, and p53 [16]. Nevertheless, the role of FXR in bladder cancer has not been investigated. In this study, we aimed to determine the effects of FXR on migration, invasion, and angiogenesis in both low- and high-grade bladder cancer cells.

## 2. Results

### 2.1. Survival Rate and Expressions of FXR in Bladder Cancer Patients and Bladder Cancer Cell Lines

**Figure 1:**
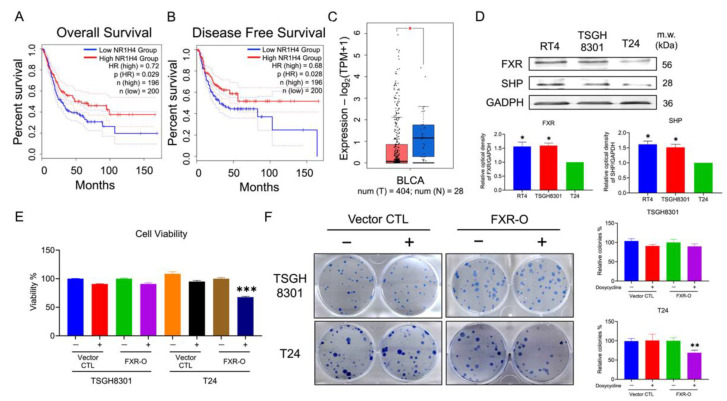
Farnesoid X receptor (FXR) expression and the effects on cell viability in human bladder cancers. (**A**,**B**) Overall and disease-free survival rate in differential expression of NR1H4 (FXR) gene in TCGA database of bladder cancer patients. (**C**) Scatter plots in differential expression of FXR gene in TCGA database of bladder cancer tissues (red plot) and adjacent normal tissues (blue plot). * *p* < 0.05 compared with the bladder cancer tissues group. (**D**) The expression levels of FXR and SHP in the bladder cancer cell lines were analyzed by Western blotting. GAPDH was a loading control. * *p* < 0.05 compared with the T24 group. (**E**) The survival rate of TSGH8301 and T24 were analyzed after doxycycline induced for 72 h in vector control and FXR overexpressed groups using MTT assay. (**F**) Colony formation assay of TSGH8301 and T24 were analyzed after 9 days of doxycycline induction in vector control and FXR overexpressed groups. Wells were visualized and quantified. ** *p* < 0.01; *** *p* < 0.001 compared with the control group.

As shown in Figure 1A–C, the relationship between the overall disease-free survival and the expression of NR1H4 (FXR) in bladder cancer tissue were analyzed by gene expression profiling interactive analysis (GEPIA). High expression of FXR in bladder cancer patient groups resulted in a higher overall and disease-free survival rate than those in low expression. Moreover, in Figure 1C, the adjacent normal tissue groups (N = 404) had higher expression of FXR than those in the bladder cancer tissue groups (T = 28). Next, the protein expression levels of FXR and its downstream target, SHP, were analyzed by Western blotting; low-grade bladder cancer cells RT4 and TSGH8301 showed higher expression in FXR and SHP (Figure 1D). These results implied that a reduction in FXR may affect the survival and malignancy in bladder cancers.

### 2.2. Overexpression of FXR Inhibited Survival and Colony Formation in T24 Cells

Doxycycline-inducible overexpression FXR and vector control systems were established in TSGH8301 and T24 cells. The intensity of protein expression of FXR were significantly increased after doxycycline induced for 48 and 72 h in both TSGH8301 and T24 cells (Supplementary Figure S1). In addition, FXR-related proteins RXR and SHP were also increased significantly after FXR overexpression groups but not in vector control groups (Supplementary Figure S1). Moreover, doxycycline had no effect on the cell viability, migration, and invasion in untransfected T24 cells (Supplementary Figure S2). Based on the above evidence, we selected doxycycline induced for 48 and 72 h in the following experiments.

MTT and colony formation assay were used to evaluate the effects of FXR overexpression on the survival rate of TSGH8301 and T24 cells. After 72 h overexpression of FXR, MTT assay showed a reduction in viability of T24 cells by 27% (Figure 1E). Moreover, the colony number was reduced by 25% in T24 cells after FXR overexpression for 9 days (Figure 1F). The above results indicated that overexpression of FXR inhibited high-grade T24 cell survival and colony-formation abilities.

### 2.3. Overexpression of FXR Inhibited the Migration and Adhesion Abilities in TSGH8301 and T24 Cells

**Figure 2:**
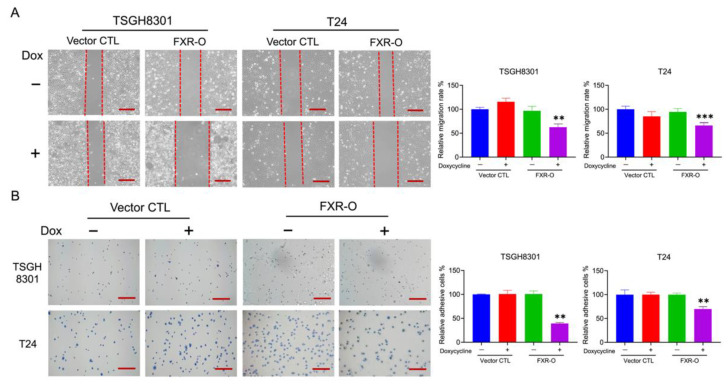
The effect on migration and adhesion abilities after FXR overexpression. (**A**) Wound healing migration assays were performed in FXR-overexpressed (FXR-O) TSGH8301 and T24 human bladder cancer cells after 6 h scratch. The right panels display the relative rate of the wound healing migratory ability. ** *p* < 0.01; *** *p* < 0.001 compared to the control group. (**B**) Adhesion assays were performed in FXR-O TSGH8301 and T24 cells after 50 min of incubation. Thereafter, the adhered cells were stained and captured. The right panels display the relative rate of the adhesive ability. ** *p* < 0.01 compared to the control group. Scale bar = 200 μm.

**Figure 3:**
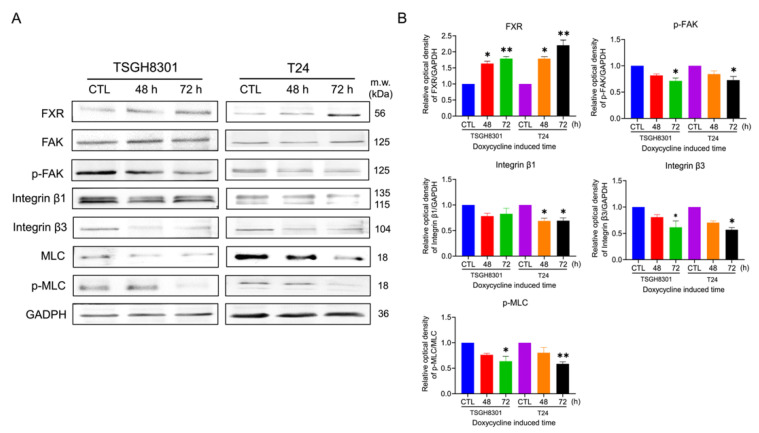
The effect on focal adhesion complex expression after FXR overexpression. (**A**) Focal adhesion kinase (FAK), phospho-focal adhesion kinase (p-FAK), integrin β1, integrin β3, myosin light chain (MLC) and phospho-myosin light chain (p-MLC) were analyzed by Western blotting in the TSGH8301 and T24 cells after FXR overexpression for 48 and 72 h. GAPDH was used as the loading control. (**B**) The bar graphs show the relative quantitative analysis of the aforementioned proteins. * *p* < 0.05; ** *p* < 0.01 compared with the control group.

The wound healing assays demonstrated that the cell migration abilities were significantly reduced by 39% and 32% in TSGH8301 and T24 cells after FXR overexpression (Figure 2A). In the adhesion assays, the adhesive ability was decreased significantly after FXR overexpression by 43% and 34% in TSGH8301 and T24 cells (Figure 2B). Moreover, the expression levels of phosphorylation of FAK, integrin β1, integrin β3 and phosphorylation of MLC were significantly reduced in both TSGH8301 and T24 cells (Figure 3). In contrast, knockdown of FXR enhanced the migration and protein expression of integrin β3 and p-MLC in low-grade RT4 and TSGH8301 cells (Supplementary Figure S3). These results suggested that FXR overexpression decreased the migration and adhesion by reducing the migratory and adhesive-related proteins in TSGH8301 and T24 cells.

### 2.4. FXR Overexpression Inhibits Migratory and Adhesive Ability via Proteosome Degradation

**Figure 4:**
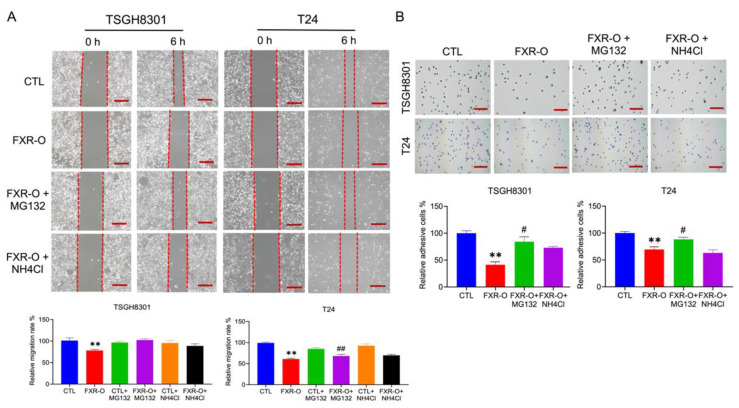
The effect of proteosome inhibitor MG132 and lysosome inhibitor NH4Cl on the migratory and adhesive abilities after FXR overexpression. (**A**) Wound healing migration assays were performed with or without MG132 or NH4Cl in TSGH8301 and T24 cells after 6 h scratch. The right panels displayed quantitative results of the relative migration rate. ** *p* < 0.01 compared to the control group; ## *p* < 0.01 compared to the FXR-O group. (**B**) Adhesion assays were performed in TSGH8301 and T24 cells for 50 min. The cells were stained and captured. The right panels displayed quantitative results of the relative adhesive rate. ** *p* < 0.01 compared to the control group; # *p* < 0.05 compared to the FXR-O group. Scale bar = 200 μm.

**Figure 5:**
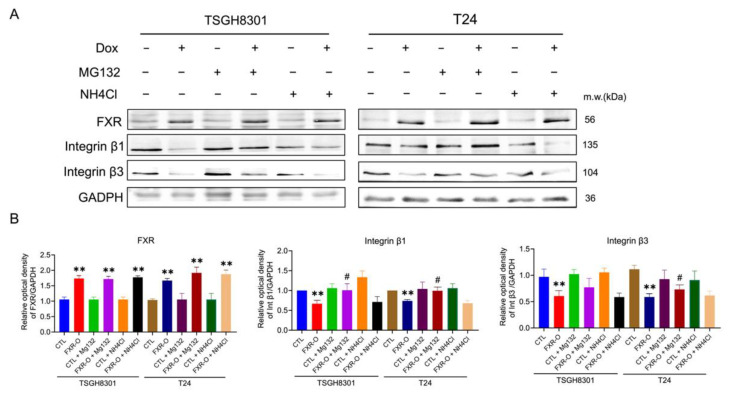
The effect of MG132 and NH4Cl on integrin expression after FXR overexpression. (**A**) Integrin β1, integrin β3, MLC and p-MLC were analyzed by Western blotting in the TSGH8301 and T24 cells with or without MG132 or NH4Cl. GAPDH was used as the loading control. (**B**) The bar graphs show the relative quantitative analysis of the aforementioned proteins. ** *p* < 0.01 compared with the control group; # *p* < 0.05 compared to the FXR-O group.

Due to the reduction in integrins after FXR overexpression, whether mRNA reduction or protein degradation occurred was investigated. The mRNA of integrin β1 and β3 was unaffected in TSGH8301 cells, while the mRNA of integrinβ3 was decreased in FXR overexpressed T24 cells (data not shown). Therefore, proteasomal and lysosomal degradation inhibitors MG132 and NH4Cl were applied to TSGH8301 and T24 cells to assess the inhibition of wound healing and adhesive abilities after FXR overexpression. Only proteosome degradation inhibitor MG132 reversed the FXR overexpression-inhibited migration and adhesion in bladder cancer cells (Figure 4). In addition, FXR overexpression-decreased integrin β1, integrin β3, and phosphorylation of MLC expression were also recovered by MG132 exposure (Figure 5). These results showed that FXR overexpression-reduced migration and adhesion of bladder cancer cells occurred through proteosome degradation.

### 2.5. Overexpression of FXR Inhibited the Invasive Ability in the T24 Cells

**Figure 6:**
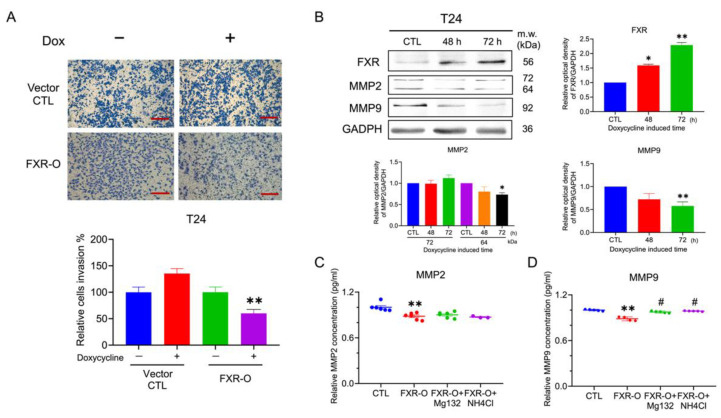
The effect of invasive abilities after FXR overexpression. (**A**) Transwell invasion assays were performed for 16 h incubation in the T24 cells after FXR overexpression. The invasive cells were stained and captured. The right panel displayed the quantitative result. ** *p* < 0.01 compared to the control group. Scale bar = 200 μm. (**B**) The expression of matrix metalloproteinases-2 (MMP2) and matrix metalloproteinases-9 (MMP9) were analyzed by Western blotting in the T24 cells. GAPDH was used as the loading control. (**C**) The concentration levels of MMP2 in the CM of T24 cells were analyzed by ELISA. (**D**) The protein activity of MMP9 in the CM of T24 cells were analyzed by the Fluorokine assay. * *p* < 0.05; ** *p* < 0.01 compared with the control group. # *p* < 0.05 compared to the FXR-O group.

Because low-grade TSGH8301 cells could not penetrate the transwell chamber with Matrigel, only T24 cells were evaluated for invasion assay. The transwell invasion assay demonstrated that the cell invasive ability was reduced by 45% in the T24 cells after FXR overexpression for 72 h (Figure 6A). Furthermore, the protein expression levels of MMP2 and MMP9 were significantly decreased (Figure 6B). Moreover, the activity of MMP2 and total MMP9 secretion were measured and separately decreased by 11% and 23% after FXR overexpression (Figure 6C,D). These results implied that the decrease in the invasive abilities after FXR overexpression may be due to the reduction of MMPs in the T24 cells.

### 2.6. Proteosome Degradation Was Involved in FXR Overexpression-Decreased Tube Formation in T24 Cells

**Figure 7:**
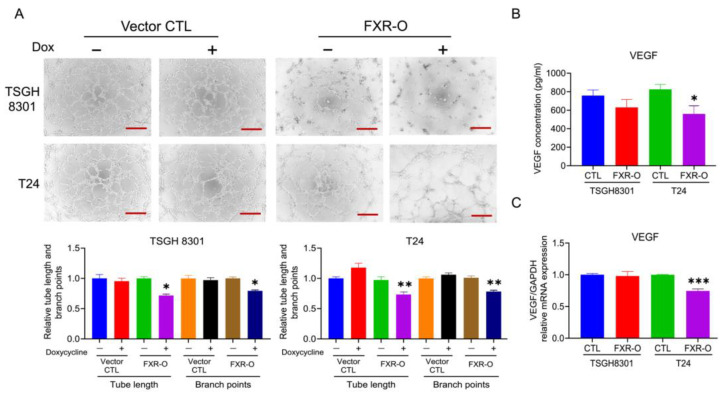
The effect of the overexpression of FXR on angiogenesis. (**A**) The human umbilical vein endothelial cells (HUVECs) were cultured with an FXR overexpression (72 h) conditioned medium (CM) and control CM of bladder cancer cells for 6 h. The formation of endothelial cell networks was observed and the number of branch points and tube length in the TSGH8301 and T24 CM were analyzed. The bar graphs show the quantitative results of relative branch points and tube lengths. Scale bar = 200 μm. (**B**) The concentration levels of vascular endothelial growth factor (VEGF) in the CM of TSGH8301 and T24 cells were analyzed by ELISA. (**C**) RT-PCR was used to analyze the mRNA expression of VEGF after FXR overexpression in TSGH8301 and T24 cells. * *p* < 0.05; ** *p* < 0.01; *** *p* < 0.001 compared with the control group.

**Figure 8:**
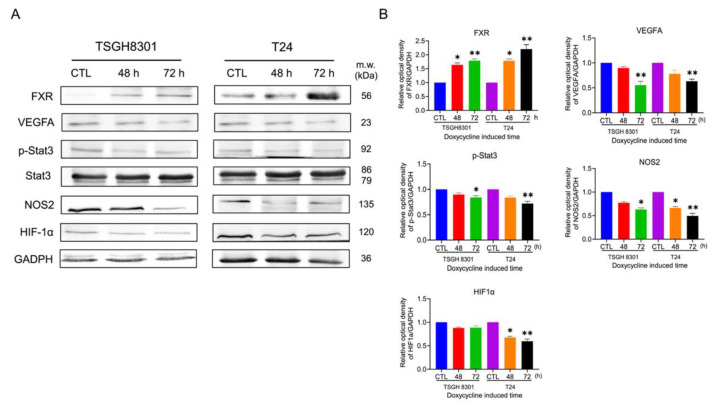
The effect of the overexpression of FXR on angiogenic related protein expression. (**A**) The expression of VEGFA, signal transducer and activator of transcription 3 (STAT3), phospho-Stat3 (p-STAT3), nitric oxide synthase 2 (NOS2) and hypoxia-inducible factors-1α (HIF-1α) were analyzed by Western blotting in TSGH 8301 and T24 cells after FXR overexpression. GAPDH was used as the loading control. (**B**) The bar graphs show the relative quantitative analysis of the aforementioned proteins. * *p* < 0.05; ** *p* < 0.01 compared with the control group.

As shown in Figure 7A, the angiogenic ability, including the branch points and tube length, was significantly decreased in the FXR overexpression groups by about 28% and 21% in TSGH8301 and 27% and 22% in T24 cells. In addition, the concentration of vascular endothelial growth factor (VEGF) in conditioned medium (CM) was reduced after FXR overexpression in T24 cells (Figure 7B). Moreover, the VEGF mRNA level was significantly reduced after FXR overexpression in T24 cells (Figure 7C). The angiogenic-related proteins including VEGFA, phosphorylated signal transducer and activator of transcription 3 (p-STAT3), nitric oxide synthase 2 (NOS2), and hypoxia-inducible factors 1α (HIF1α) were significantly reduced in both TSGH8301 and T24 cells (Figure 8). Applying both VEGF121 and VEGF165 reversed the tube-formation ability in FXR overexpression groups in T24 cells (Supplementary Figure S4). These results indicated that the angiogenic ability of HUVECs was inhibited by FXR overexpression in the TSGH8301 and T24 cells, which resulted from the decreased VEGF secretion, mRNA expression, and angiogenic- related protein expression in human bladder cancer cells.

**Figure 9:**
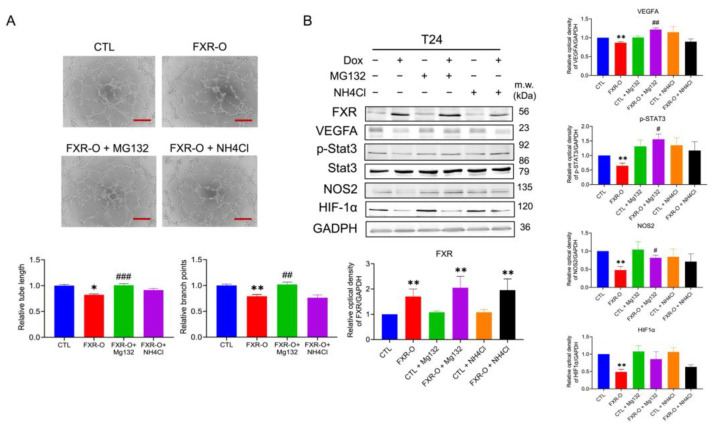
The effect of proteosome inhibitor MG132 and lysosome inhibitor NH4Cl on the angiogenic abilities after FXR overexpression. (**A**) The human umbilical vein endothelial cells (HUVECs) were cultured with FXR overexpressed conditioned medium (CM) and control CM with or without MG132 or NH4Cl of bladder cancer cells for 6 h. The formation of an endothelial cell network was observed and the number of branch points and tube length in the T24 CM were analyzed. Scale bar = 200 μm. (**B**) VEGFA, p-STAT3, NOS2 and HIF-1α were analyzed by Western blotting in T24 cells with or without MG132 or NH4Cl. GAPDH was used as the loading control. The bar graphs show the relative attenuation of branch points and tube lengths. * *p* < 0.05, ** *p* < 0.01 compared with control group; # *p* < 0.05, ## *p* < 0.01; ### *p* < 0.001 compared with FXR overexpression group.

Next, proteasomal and lysosomal degradation inhibitors MG132 and NH4Cl were added to assess the inhibition of tube-formation abilities after FXR overexpression in T24 cells. Only MG132 reversed the angiogenic abilities after FXR overexpression in T24 cells (Figure 9A). Additionally, MG132 exposure significantly reversed VEGFA and NOS2 expression, which were reduced by FXR overexpression (Figure 9B). These results showed that proteasomal degradations were involved in the FXR overexpression-inhibited angiogenic ability of bladder cancer cells.

### 2.7. HUVECs Migratory Abilities Were Reduced by Proteasomal Degradation

**Figure 10:**
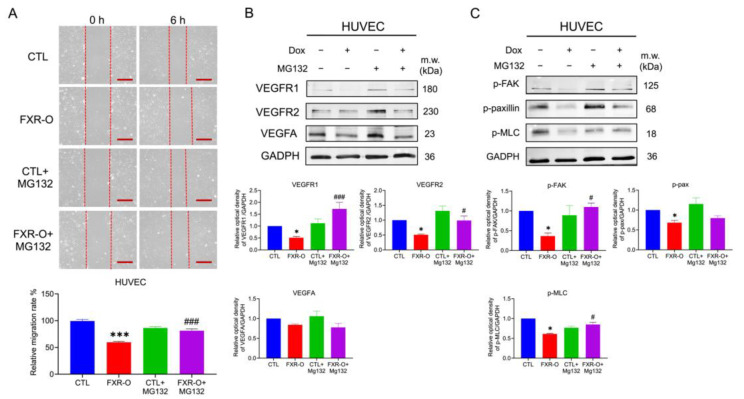
The effect of proteosome inhibitor MG132 on the migration after FXR overexpression CM in HUVECs. (**A**) Wound healing migration assays were performed in HUVECs after treatment with FXR overexpression CM with or without MG132. The lower panel displays quantitative results of the relative migration rate of the wound healing migratory abilities. *** *p* < 0.001 compared to the control group; ### *p* < 0.001 compared to the FXR-O group. Scale bar = 200 μm. (**B**,**C**) VEGFR1, VEGFR2, VEGFA, p-FAK, p-paxillin, p-MLC were analyzed by Western blotting in HUVECs with or without MG132. GAPDH was used as the loading control. The bar graphs show the relative quantitative analysis of the aforementioned proteins. * *p* < 0.05 compared with the control group; # *p* < 0.05; ### *p* < 0.001 compared to the FXR-O group.

The wound-healing assays demonstrated that supplementation with FXR overexpression CM decreased HUVECs migration (Figure 10A). The protein expression of VEGFR1, VEGFR2, VEGFA, p-FAK, and p-MLC were downregulation in HUVECs when cultured with FXR overexpression CM (Figure 10B). Moreover, the proteosome inhibitor MG132 restored the migration inhibition and the aforementioned protein expression in HUVECs (Figure 10). These results suggested that the migratory abilities of HUVECs reduced by FXR overexpression CM were also mediated by proteasomal degradation.

## 3. Discussion

FXR overexpression reduced the cell survival ability in T24 cells. In esophageal squamous cell carcinoma, the FXR agonist GW4064 impaired esophageal squamous cell carcinoma proliferation and migration by inducing apoptosis and cell-cycle arrest and suppressing phosphorylation of ERK1/2 protein expression [17]. In human hepatocellular carcinoma, FXR overexpression significantly represses liver cancer cell proliferation, and tumor growth in nude mice resulted in a marked increase of SHP expression [18]. In cervical cancer, overexpression of FXR induced early and late apoptosis and promoted G1 arrest through upregulation of SHP, MDM2, and p53 [16]. In liver cancer, FXR overexpression suppresses proliferation of human liver cancer cells via the inhibition of the mTOR/S6K signaling pathway. These results suggest that decreased FXR viability in human bladder cancer cells might result from the decrease in proliferation.

The overexpression of FXR inhibited the migration and adhesion abilities in human bladder cancer cells. Previous studies showed that integrin binding to ECM results in the phosphorylation of paxillin and FAK, giving rise to increased cells adhesion and migration [19,20,21,22]. In liver cancer SK-Hep-1 cells and colorectal cancer, FXR suppresses migration by suppressing the Wnt/β-catenin signaling pathway [23,24]. Furthermore, the Wnt/β-catenin signaling pathway is linked to the activation of integrin β1 to drive migration in glioma cells [25]. In colon cancer, the knockdown of FXR increased the migration of colon cancer cells by inducing the protein expression of EMT markers such as vimentin, snail, slug, fibronectin, and FAK [24]. In our results, FXR overexpression decreased integrin β1, integrin β3, p-FAK and p-MLC expression, which resulted in the downregulation of migratory and adhesive abilities in bladder cancer cells.

The proteosome inhibitor MG132 reversed the FXR overexpression-inhibited adhesive ability both in TSGH8301 and T24 cells. MG132 has been reported to enhance migration and mesenchymal phenotype in A549 lung cancer cells [26]. Moreover, MG132 recovers phospholipase C-γl overexpression reduced adhesion and migration in rat fibroblasts [27]. Nevertheless, in migration, MG132 could significantly reverse the FXR overexpression-inhibited migratory ability only in T24 cells. Previously, it has been reported that integrin β1 mainly regulates cell adhesion but integrin β3 largely regulates cell migration [28]. Our results indicated that integrin β1 declined both in TSGH8301 and T24 cells; however, integrin β3 only decreased significantly in T24 cells. These results implied that a different effect of MG132 on migration and adhesion in TSGH8301 and T24 cells. Taken together, FXR overexpression reduced the expression of migratory-related proteins, which may lead to proteosome degradation in bladder cancer cells.

The expression of MMP2 and MMP9 was significantly reduced after FXR overexpression, which may inhibit invasion in human bladder cancer T24 cell. MMPs mediate the breakdown of the basal membrane, degrade the extracellular matrix, and create a microenvironment that enhances tumor cell migration and invasion [15]. In colon cancer, FXR overexpression inhibits colon cancer cell proliferation and invasion in vitro by suppressing MMP7 mRNA and protein expression [29]. In liver cancer, FXR overexpression results in a significant reduction in the total and nuclear β-catenin proteins and its downstream target genes, including c-Jun and MMP9, in vitro and in vivo that contribute to the inhibition of cell invasive abilities. Therefore, MMP9 protein was decreased in human bladder cancer T24 cells, which might be due to β-catenin reduction. However, after being treated with proteosome inhibitor MG132 and lysosome inhibitor NH4Cl, neither of the above can reverse the FXR overexpression-inhibited invasion in T24 cells (data not shown). Although both MG132 and NH4Cl could reverse the activity of MMP9, they could not prevent the reduction in MMP2 (Figure 6C,D). The mechanism of FXR overexpression inhibited invasion, MMP2 and MMP9 expression may need further investigation.

Overexpression of FXR inhibited the angiogenesis ability via VEGF reduction in endothelial cells. VEGFA is an abundant effector molecule that is secreted from tumors, mesothelial cells and inflammatory cells, and contributes to vascular formation [30]. For tumor growth and metastasis, growth of the vascular network is essential to supply nutrients and oxygen and also remove waste products [31]. Moreover, hypoxia activates hypoxia-inducible transcription factors (HIFs), which induces the expression of angiogenic factors including VEGFs and NOS [32]. In hepatocellular carcinoma, STAT3 activated by LPS increases the production of VEGF by tumor cells, which promotes the proliferation and angiogenesis of HCC cells [33]. The mRNA expressions of VEGF, VEGFR2, PI3K and p38 downregulate significantly while FXR is overexpressed in pulmonary fibrosis [34]. In our results, FXR overexpression significantly reduced the VEGF and iNOS mRNA level in T24 cell and protein expression of VEGFA, HIF1α, p-STAT3 and iNOS in both TSGH8301 and T24 cells. Treatment with the proteosome inhibitor MG132 in T24 cells reversed both the tube formation abilities and angiogenic-related proteins, as well as VEGFA, HIF1α, p-STAT3 and iNOS. Therefore, FXR overexpression may enhance proteasomal degradation, leading to VEGFA, p-STAT3 and HIF1α down-regulation and reduced angiogenesis.

The inhibition of migratory abilities in HUVECs cultured with FXR overexpression CM was observed. Cancer–endothelial cell interactions in the tumor microenvironment cause the secretion of adhesion molecules and chemokines, which are critical to tumor growth and metastasis [35]. Previous studies have shown that the integrin-induced signal pathway is involved in endothelial cell migration, and the integrins-FAK-Rho GTPases are activated in both endothelial and cancer cells [36]. Cancer cells secrete growth factors, including VEGF, COX-2 and NF-κB, which significantly increase endothelial cell proliferation, migration, and tube formation [37]. In breast cancer, angiotensin-converting enzyme 2 inhibited endothelial cells proliferation, tube formation, and migration through the phosphorylation of VEGFR2, MEK1/2, and ERK1/2 in HUVECs through the downregulation of VEGFA in breast cancer cells [38]. Moreover, MG132 abrogates a reduction in VEGFR2, AKT, and ERK1/2 caused by octaminomycins in HUVECs [39]. As in our results, FXR overexpression CM significantly decreased the proteins expression of VEGFA, VEGFR1, VEGFR2, p-FAK, and p-MLC in HUVECs, which could be blocked by MG132 treatment. FXR overexpression in bladder cancer cells contributed to migration reduction in HUVECs through proteasomal degradation, as well as a decrease in VEGFR, VEGFA, p-FAK and p-MLC expression.

In our studies, FXR overexpression inhibited bladder cancer cell migration, adhesion, and angiogenesis in human bladder cancer cells TSGH8301 and T24 through proteasome degradation pathway. However, there are still some limitations that need to be improved. First, although the impressive effects of FXR on bladder cancer cells were found in our in vitro experiments, the effects of FXR in animal models represent an issue that still needs to be solved. Second, the invasive ability and its related proteins MMP2 and MMP9 cannot be reversed in both MG132 and NH4Cl. The relationship between FXR overexpression and invasion inhibition in muscle invasive human bladder cancer T24 also needs further investigation. Third, the FXR agonist obeticholic acid has been demonstrated to have promising clinical results in the treatment of liver disorders such as primary biliary cirrhosis, primary sclerosing cholangitis, and nonalcoholic steatohepatitis [40]. However, the effects of FXR agonist on bladder cancer is still unknown and needs additional investigation.

## 4. Materials and Methods

### 4.1. Cell Culture

Three different grades of bladder cancer cell lines (Tri-Service General Hospital 8301 (TSGH8301)) were provided from the Division of Urology, Tri-Service General Hospital, National Defense Medical Center, and incubated in RPMI 1640 medium. RT4 and T24 cells were obtained from the Bioresource Collection and Research Center (BCRC), Taiwan, and grown in McCoy’s 5a medium. All mediums were supplemented with 10% fetal bovine serum (FBS) (Thermo Fisher Scientific, Waltham, MA, USA), 1% L-glutamine, and 1% sodium pyruvate (Corning, NY, USA). Human umbilical vein endothelial cells (HUVECs) were purchased from the BCRC in Taiwan and cultured in endothelial cell media (ECM) (ScienCell Research Laboratories, Carlsbad, CA, USA). Cells were all incubated in 5% CO_2_ at 37 °C.

### 4.2. Plasmid Construction, Lentivirus Production and Doxycycline Induciable Overexpression

The FXR cDNA transcript variant 1 clone and the P2A-DsRed sequence synthesis were provided from GenScript Biotech (Piscataway, NJ, USA). The FXR-P2A-DsRed was cloned into the pAS4.1w.Puro-aOn, a tetracycline-inducible plasmid. The lentivirus was produced by 293T cells co-transfected with the lentiviral vectors FXR-P2A-DsRed::pAS4.1w.Ppuro-aOn, pCMV-dR8.91, and pMD2.G using Lipofectamine 3000 reagent (Thermo Fisher Scientific, Waltham, MA, USA) according to the manufacturer’s instructions. The National RNAi Core Facility provided the pAS4.1w.Puro-aOn and lentivirus package plasmids at Academia Sinica in Taiwan. The lentivirus was used to infect TSGH8301 and T24 cells. All cells were selected by puromycin (2 μg/mL). After antibiotics selection, the cells were sorted by FACSAria IIIu sorter (BD, Franklin Lakes, NJ, USA). The protein expression of FXR was checked after doxycycline was induced for 48 and 72 h.

### 4.3. MTT Assays

TSGH8301 and T24 human bladder cancer cells were seeded in 96-well plates at a density of 2 × 10^3^ cells (TSGH8301) or 1 × 10^3^ cells (T24) per well. Doxycycline (1 μg/mL) was added to the FXR overexpression groups for 72 and 96 h. Next, MTT reagent (3-(4,5-dimethyl-2-thiazolyl)-2,5-diphenyl-2H-tetrazolium bromide, Sigma-Aldrich, Brulington, MA, USA) was added, and after 3 h of incubation, MTT reagent was removed and cells were lysed and measured at an absorbance of 590 nm.

### 4.4. Colony Formation Assays

Fifty TSGH8301 and 100 T24 human bladder cancer cells were cultured in six-well plates. Cells were treated with or without culture medium with doxycycline (1 μg/mL) and the mediums were changed every two days. After nine days of culture, the cells were fixed with 10% paraformaldehyde and stained with Coomassie Brilliant Blue G250 (Sigma-Aldrich, Brulington, MA, USA). Then, the colonies in each well were counted.

### 4.5. Wound Healing Migration Assay

TSGH8301 and T24 human bladder cancer cells or HUVECs were seeded and separated into four groups: control (CTL), FXR overexpression (FXR-O), FXR overexpression with MG132 (200 ng/mL) (FXR-O+MG132) and FXR overexpression with NH4Cl (200 ng/mL) (FXR-O+NH4Cl). MG132 and NH4Cl were treated for 24 h before the experiments. Wounds were scratched by a 200 μL pipette tip. The migratory ability in TSGH8301, T24, and HUVEC cells was analyzed after various treatments for 6 h. Then, the wound images were captured and the areas were analyzed by ImageJ.

### 4.6. Adhesion Assays

The six-well plates were prepared and coated with fibronectin (1 μg/mL) in a 37 °C incubator for 15 min. After various treatments, the cells were suspended in culture medium and seeded into wells precoated with fibronectin. After incubation at 37 °C for 50 min, the plates were washed with PBS to remove the non-adherent cells. The adhesive cells were fixed and stained. Cells were examined in three randomly selected fields from each well. The fields were then captured and analyzed.

### 4.7. Transwell Assays

T24 cells were seeded in the upper chamber of a Transwell at a density of 3 × 10^4^ cells/well (Corning Costar, Midland, NC, USA). Before seeding, 3% Matrigel mixed with McCoy’s 5A medium was added to the upper chamber and incubated for 2 h at 37 °C. After incubating at 37 °C for 16 h, the cells on the lower chamber were fixed and stained. Invading cells were captured and analyzed in three randomly selected fields from each transwell.

### 4.8. Tube Formation Assays

Matrigel (50 mL/well) was added to a precooled 96-well plate and incubated for 2 h at 37 °C. HUVECs (1 × 10^4^) were seeded into each well with 50% conditioned medium (CM). After 6 h of incubation, the tube formation was imaged. Then, the tube length and branch points were quantified and analyzed by AngioTool.

### 4.9. Real-Time Polymerase Chain Reaction (RT-PCR)

After various treatments, the cells were collected by GENEzol™ Reagent and the total RNA was purified by the GENEral™ TriRNA Pure Kit from Geneaid in Taiwan. The RNA was reverse-transcribed to cDNA by the PrimeScript™ RT reagent kit (TAKARA Bio Inc., Shiga, Japan) according to the manufacturer’s instructions. RT-PCR was carried out with a LightCycler^®^ 480 Instrument (Roche, Basel, Switzerland) using SensiFAST SYBR (Meridian Bioscience, Cincinnati, OH, USA). The oligonucleotide primers used are listed in Supplementary Table S1. The GAPDH gene was used as an internal control.

### 4.10. Western Blotting

After different treatments, the cells were collected with mammalian protein extraction buffer (GE Healthcare Life Sciences, Chicago, IL, USA) containing proteinase inhibitor and phosphatase inhibitor (MedChem Express Monmouth Jucntion, NJ, USA). Different protein samples were electrophoresed on a 10% or 11% SDS-PAGE and then transferred to a nitrocellulose membrane (Bio-Rad, Berkeley, CA, USA). The membranes were blocked and incubated with primary antibodies at 4 °C overnight. After being washed, the strips were incubated with a 1:5000 dilution of HRP-conjugated anti-rabbit or anti-mouse IgG antibody from Cell Signaling Technology at room temperature for 1 h. Next, the blots were reacted with the ECL substrate developing solution (Bio-Rad, Berkeley, CA, USA) and protein signals were detected by Xplorer (SPOT Imaging, Sterling Heights, MI, USA). The density of the bands on the nitrocellulose membrane (Bio-Rad, Berkeley, CA, USA) was quantified and analyzed using ImageJ. The density of the control samples was designated as 100%, and the density of the test sample was then obtained relative to the density of the control sample. The antibodies used are listed in Supplementary Table S2. 

### 4.11. Enzyme-Linked Immunosorbent Assay (ELISA) for VEGF and MMP2

The concentration of VEGF and MMP2 in the conditioned medium was measured by the ELISA Kit (R&D Systems, Minneapolis, MN, USA and abcam, ab100606, Cambridge, MA, USA) according to the manufacturer’s instructions. The values detected by ELISA were corrected using a dilution factor and expressed in picograms per milliliter (pg/mL).

### 4.12. Human Active MMP9 Fluorokine Eassay

The active form of MMP9 was measured by the Fluorokine Human MMP9 Kit (R&D Systems, Minneapolis, MN, USA) according to the manufacturer’s instructions.

### 4.13. Statistical Analysis

The overall survival data sets obtained from The Cancer Genome Atlas (TCGA) database were analyzed using the Kaplan–Meier method on the Gene Expression Profiling Interactive Analysis (GEPIA) website. All results included at least five independent experiments. The results are presented as the mean ± standard error of the mean (SEM). Differences were analyzed using the Kruskal–Wallis test. Post hoc analysis was performed using the Mann–Whitney test. Statistical significance was set at *p* < 0.05.

## 5. Conclusions

**Figure 11:**
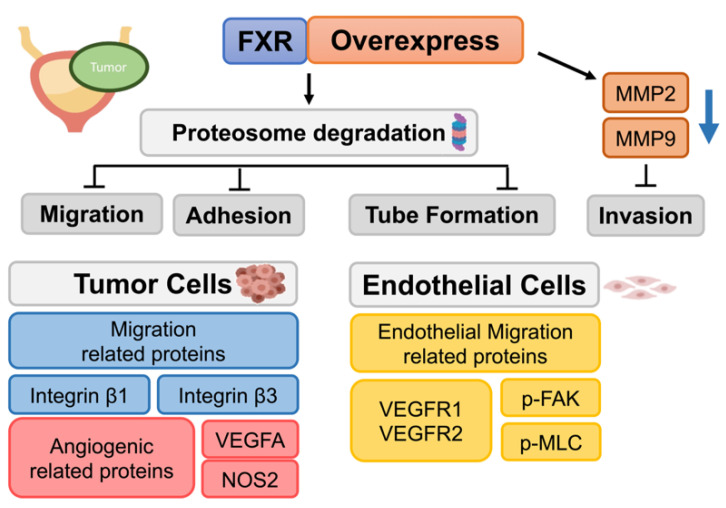
Scheme of FXR overexpression downregulated bladder cancer cell migration, adhesion, and tube formation.

This is the first study to investigate the role of FXR in human bladder cancer. In our study, the overexpression of FXR resulted in the inhibition of the migration, adhesion, and angiogenesis in human bladder cancer cells. Moreover, FXR overexpression reduced the secretion of angiogenic-related factor VEGFA and endothelial-cell-migration-related proteins VEGFR1, VEGFR2, p-FAK, and p-MLC, leading to the declining effects of HUVEC angiogenesis and migration. Finally, the proteosome inhibitor MG132 may reversed the effects of FXR. These findings indicated that FXR may serve as a potential target for therapeutic strategies in human bladder cancer in the future (Figure 11).

## Data Availability

The data presented in this study are available on request from the corresponding author. Data may be available upon request to interested researchers.

## Supplementary Materials

The following supporting information can be downloaded at: https://www.mdpi.com/article/10.3390/ijms23095259/s1.
