# Supplementary material for: Enhancement of Farnesoid X Receptor Inhibits Migration, Adhesion and Angiogenesis through Proteasome Degradation and VEGF Reduction in Bladder Cancers
Source: Int J Mol Sci. 2022 May 9;23(9):5259. doi: 10.3390/ijms23095259 (PMC9103877; doi:10.3390/ijms23095259)
Supplement: Supplementary file 1 [file ijms-23-05259-s001.zip › ijms-1716245-supplementary.pdf]

International Journal of
Molecular Sciences

MDPI

Supplementary Figures

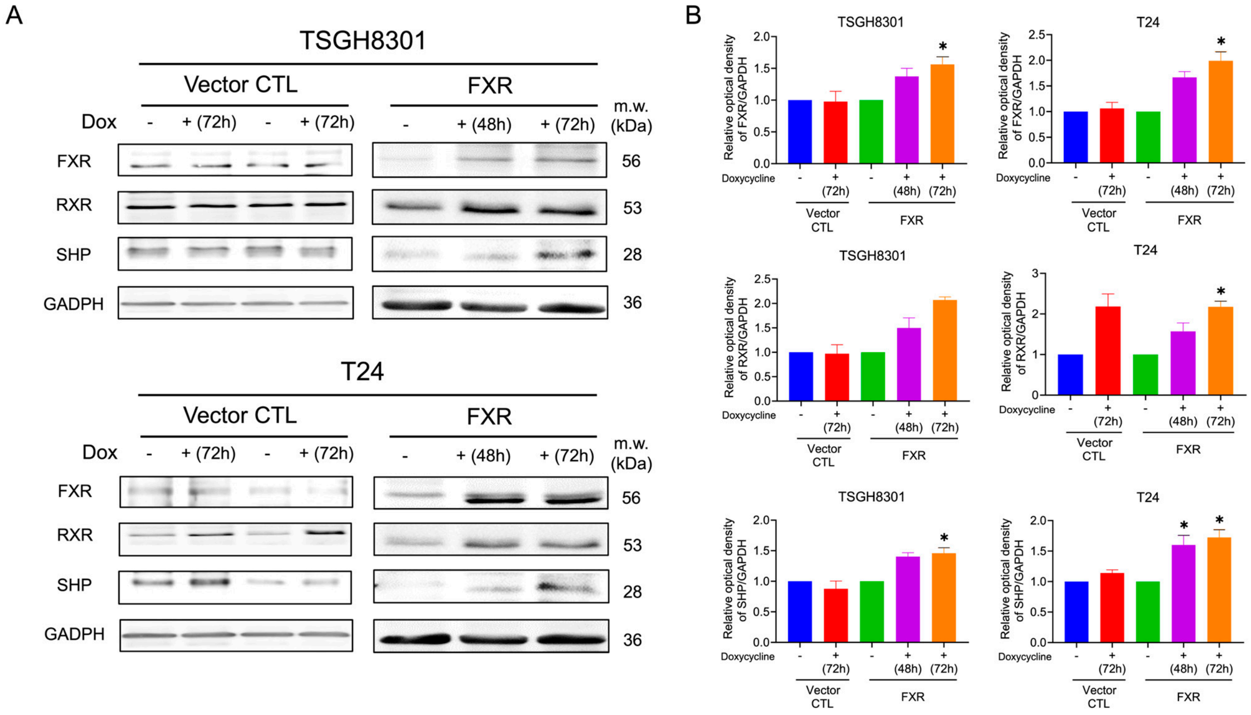

Supplementary Figure S1. Inducible FXR overexpression system in human bladder cancer cells TSGH8301 and T24. (**A**) TSGH8301 cells were treated with 1 mg/ml doxycycline for 48 to 72 h and analyzed FXR, retinoid X receptor (RXR) and small heterodimer partner (SHP) protein expressions by western blotting. GAPDH was as a loading control. (**B**) The bar graphs show the relative quantitative analysis of the aforementioned proteins. * $p < 0.05$ compared with the control group.

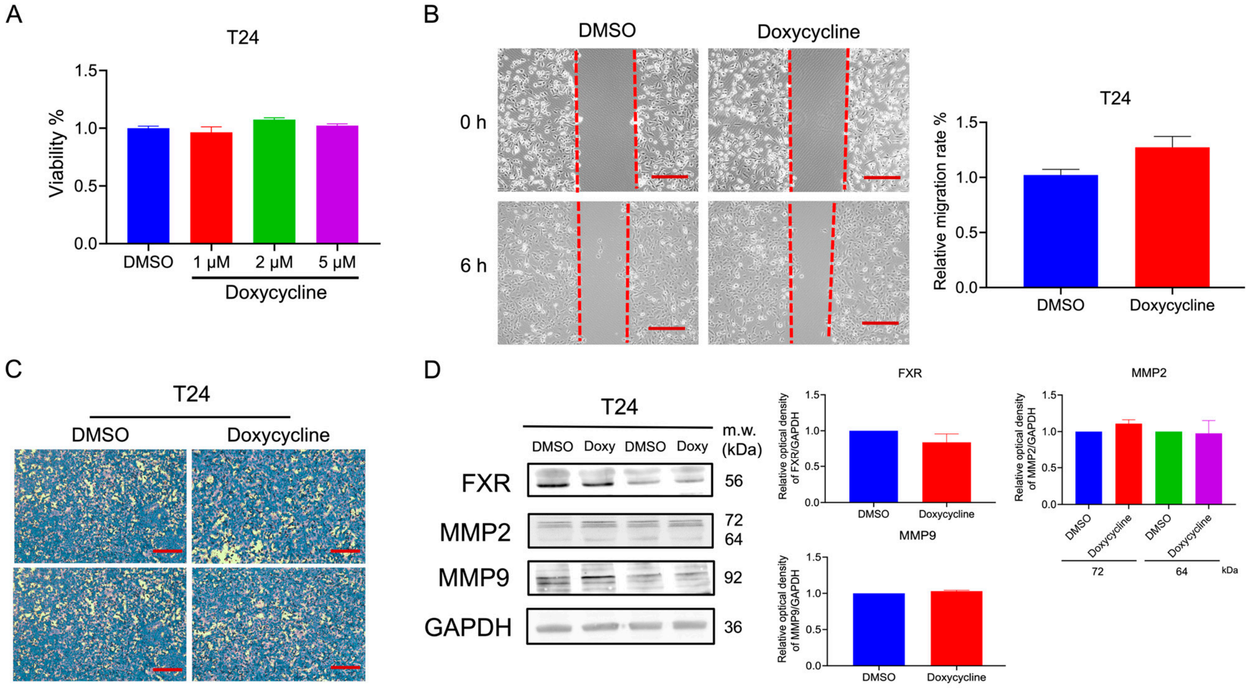

Supplementary Figure S2. The effect of doxycycline on cell viability, migration and invasion. (**A**) The cell viability of T24 cells was analyzed after doxycycline treatment for 72 h by MTT assay. (**B**) Wound healing migration assays were performed after doxycycline treatment for 72 h with 6 h scratch. The right panel display the relative rate of the wound healing migratory ability. (**C**) Transwell invasion assays were performed for 16 h incubation in the T24 cells after 72 h doxycycline treatment. The invasive cells were stained and captured. (**D**) The expression of FXR, matrix metalloproteinases-2 (MMP2) and MMP9 were analyzed by Western blotting in the T24 cells. GAPDH was used as the loading control.

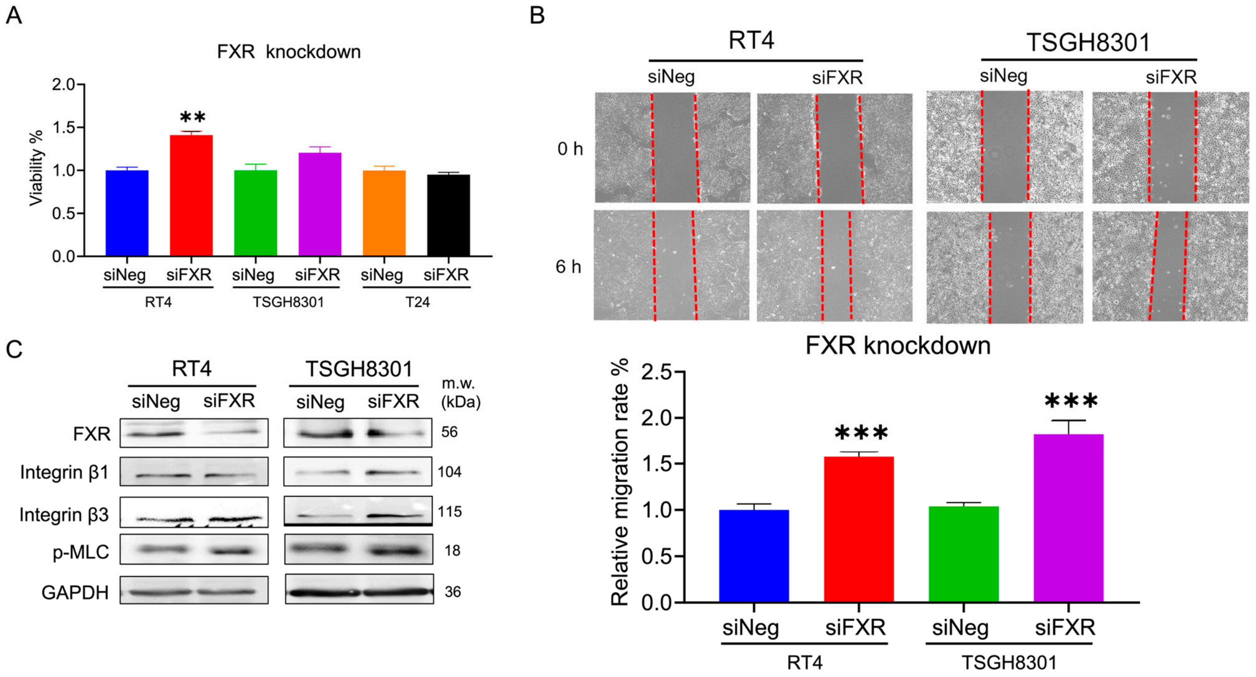

Supplementary Figure S3. The effect of FXR knockdown on cell viability and migration. (**A**) The survival rate of RT4, TSGH8301 and T24 cells were analyzed after FXR knockdown for 72 h by MTT assay. (**B**) Wound healing migration assays were performed in FXR knockdown (siFXR) RT4 and T24 human bladder cancer cells after 6 h scratch. The lower panels display the relative rate of the wound healing migratory ability. *** $p < 0.001$ compared to the control group. (**C**) FXR, integrin β1, integrin β3, and phospho-myosin light chain (p-MLC) were analyzed by Western blotting in the RT4 and T24 cells after FXR knockdown for 72 h. GAPDH was used as the loading control.

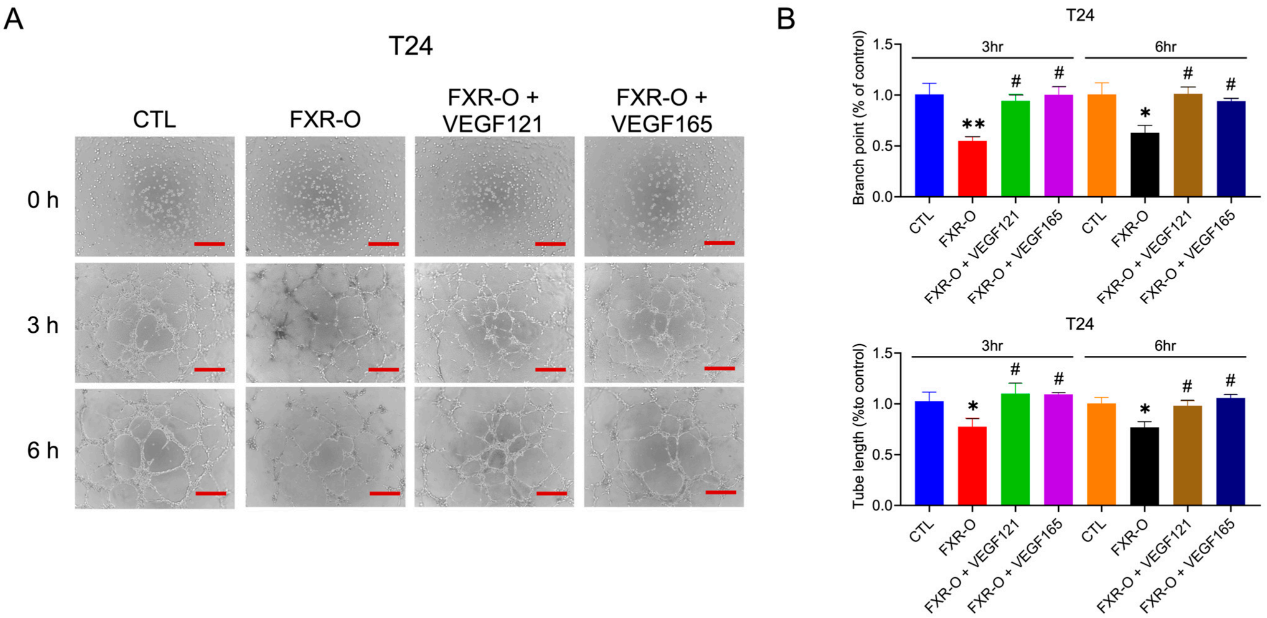

Supplementary Figure S4. The effect of VEGFA application on tube formation in FXR overexpression T24 cells. (**A**) Conditioned medium (CM) was collected after 72 h cell culture incubation. Tube formation assay was performed with the addition of VEGFA121 (400 pg/mL) and VEGFA165 (400 pg/mL) in FXR overexpression group. Total length of HUVECs were captured and measured after 6 h incubation. (**B**) The bar graphs show the branch points and tube lengths. * $p < 0.05$; ** $p < 0.01$ compared with the control group. # $p < 0.05$ compared with FXR overexpression group. Scale bar = 200 µm

Supplementary Table S1. Primer lists.

Name	Sequence
GAPDH-QF	CCA CAT CGC TCA GAC ACC AT
GAPDH-QR	TGA CCA GGC GCC CAA TA
FXR-QF	GACTTTGGACCATGAAGACCAG
FXR-QR	GCCCAGACGGAAGTTTCTTATT
Integrin β1-QF	GTAACCAACCGTAGCAAAGGA
Integrin β1-QR	TCCCCTGATCTTAATCGCAAAAC
Integrin β3-QF	CATGAAGGATGATCTGTGGAGC
Integrin β3-QR	AATCCGCAGGTTACTGGTGAG
VEGFA-QF	AGGGCAGAATCATCACGAAGT
VEGFA-QR	AGGGTCTCGATTGGATGGCA

Supplementary Table S2. The information of antibody.

Name	Species	Brand	Cat NO.
GAPDH	Rb	CST	5174
FXR	Ms	SANTA CRUZ	sc-25309
SHP	Ms	SANTA CRUZ	sc-271511
RXRα	Rb	CST	3085
p-FAK	Ms	BD	611723
FAK	Rb	CST	71433
Integrin β1	Rb	CST	9661S
Integrin β3	Ms	CST	9746S
p-MLC	Rb	abcam	ab200809
MLC	Rb	abcam	ab92721
MMP2	Rb	CST	13132
MMP9	Rb	CST	13667
VEGFA	Ms	abcam	ab1316
VEGFR1	Rb	abcam	ab32152
VEGFR2	Rb	CST	2479S
p-STAT3	Ms	BD	612542
STAT3	Rb	CST	4904S
NOS2	Ms	SANTA CRUZ	sc-7271
HIF 1α	Ms	Novus	NB100-105
